# Supplementary figures and images for: Constitutive expression and distinct properties of IFN-epsilon protect the female reproductive tract from Zika virus infection
Source: PLoS Pathog. 2023 Mar 10;19(3):e1010843. doi: 10.1371/journal.ppat.1010843 (PMC10032502; doi:10.1371/journal.ppat.1010843)

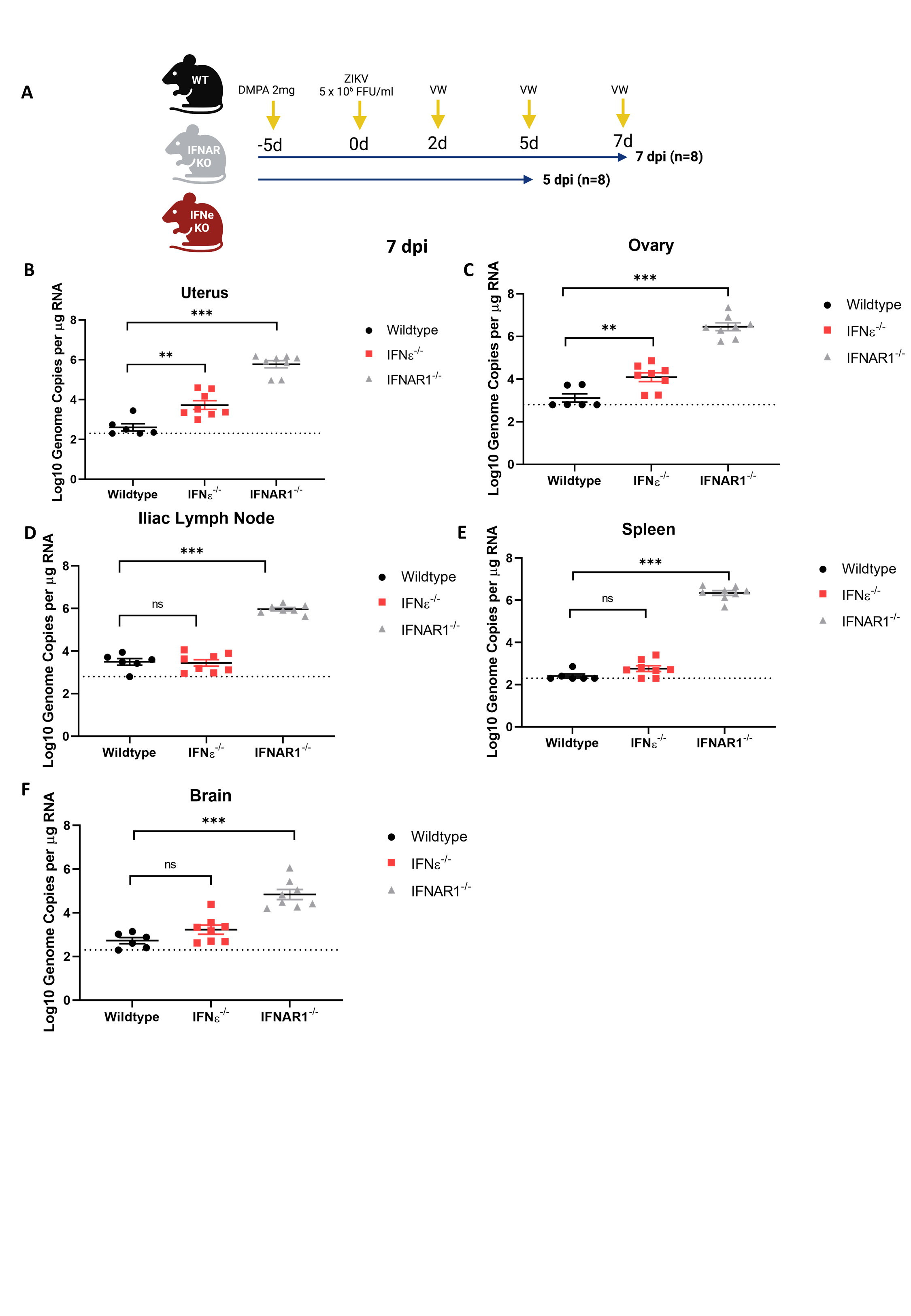

Supplement: S1 Fig — A) Experimental timeline of WT (black), mice lacking IFNε (IFNε-/-) (red) or mice lacking the type-I IFN receptor (IFNAR1-/-) (grey) were infected with ZIKV at 5 X10^5 FFU 5 days post DMPA treatment and vaginal washes were taken at 2, 5, 7 dpi. 8 mice were culled at 5 dpi and 8 were culled at 7 dpi. B, C, D, E, & F) Tissues taken at 7 dpi were used to harvest RNA for analysis of viral RNA by qRT-PCR in the uterus, ovary, illiac lymph node spleen and brain respectively. Schematic created with BioRender.com. (TIFF) [file ppat.1010843.s001.tiff]

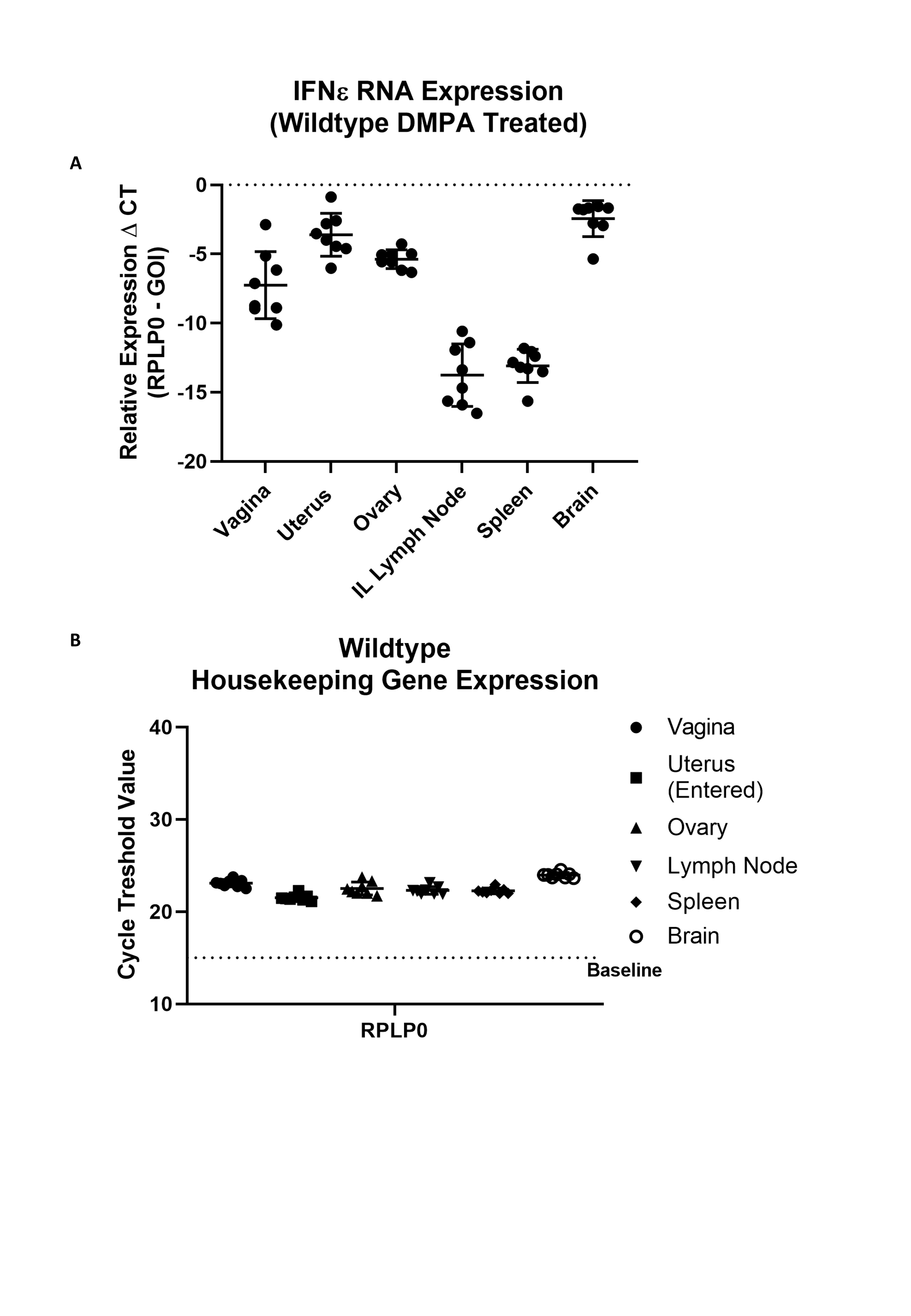

Supplement: S2 Fig — A) Uninfected mice (n = 8) were culled 10 days post DMPA treatment to mirror the timeline of the experiment shown in Fig 1 and tissues were collected as previously described. A) the level of IFNɛ RNA was determined by qRT-PCR and was expressed as ΔCT normalised to expression of the housekeeping gene RPLP0 (36B4). B) Expression of RPLP0 was compared between the tissues by qRT-PCR and expressed as raw cycle threshold value. (TIF) [file ppat.1010843.s002.TIF]

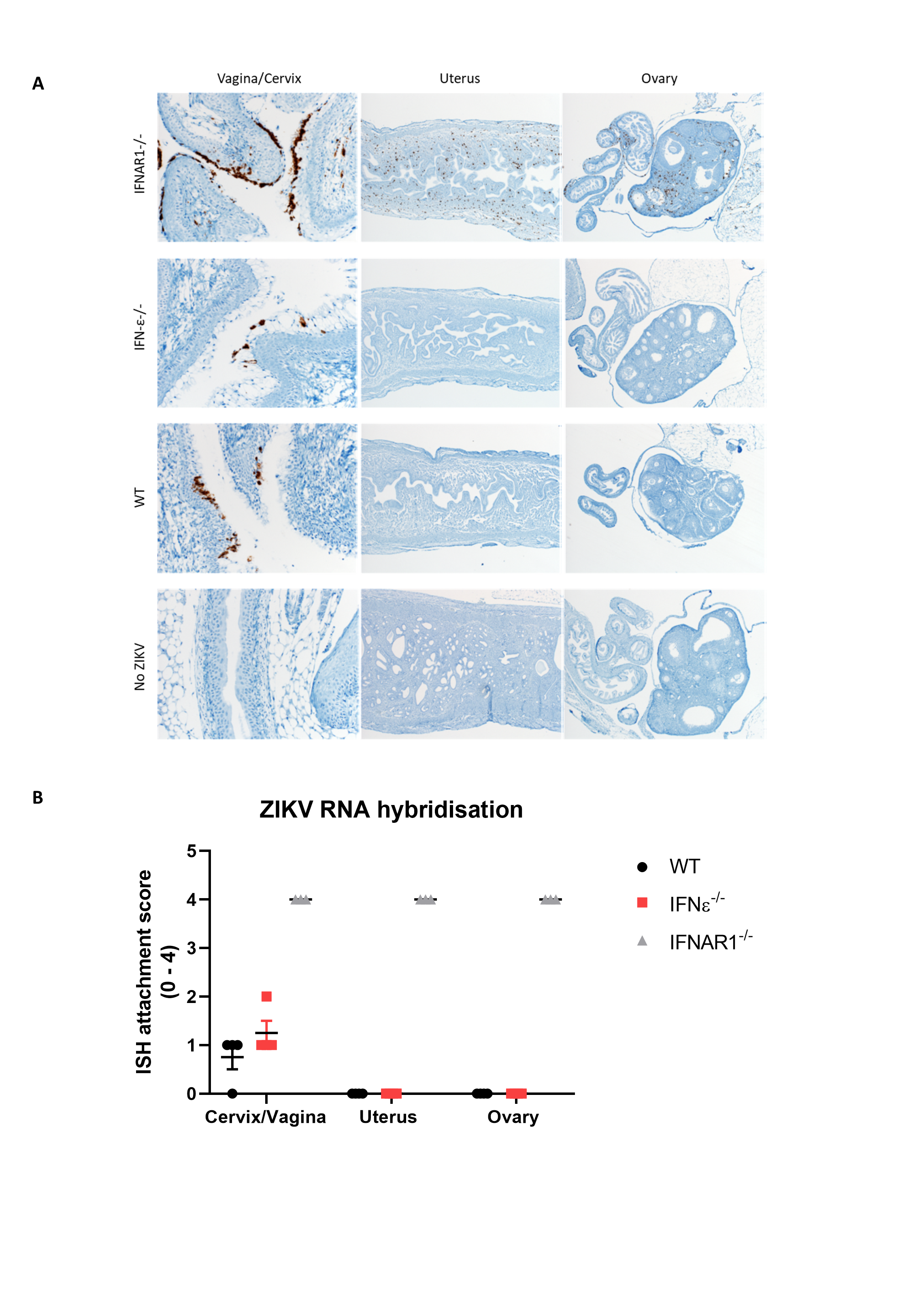

Supplement: S3 Fig — Whole FRT tissues (vagina, cervix, uterus and ovary) were fixed in formalin for 24 h prior to paraffin embedding and sectioning (5 μM), The RNAscrope protocol was used to the manufacturers specifications to detect ZIKV infection using a probe specific to ZIKV–ssRNA. A) Representative ISH images detecting from WT, IFNε-/- and IFNAR-/- mice (ZIKV in brown). B) Pathologist scoring of ISH attachment (0 = none, 1 = rare/few, 2 = scattered, 3 = moderate, 4 = numerous). (TIF) [file ppat.1010843.s003.TIF]

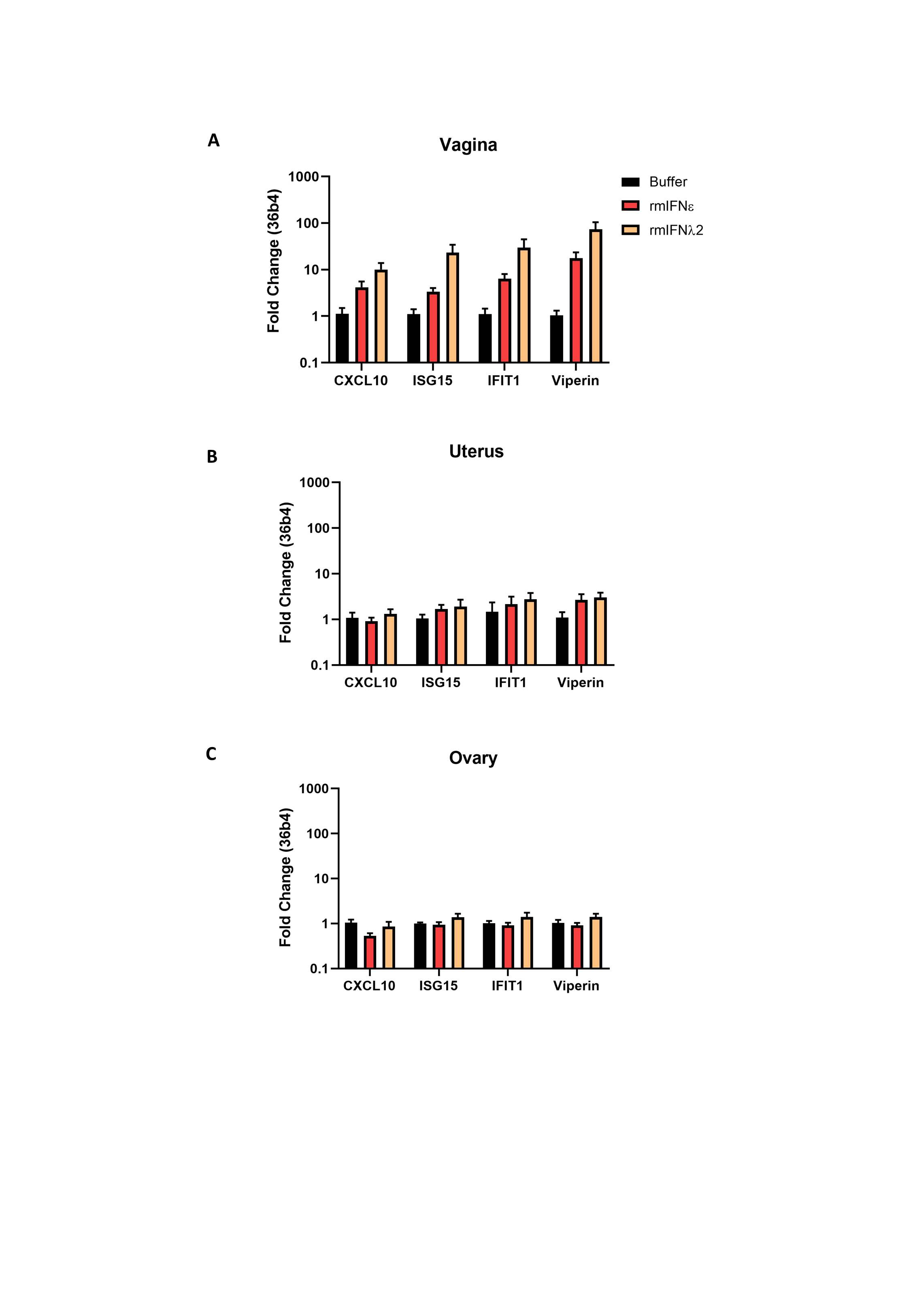

Supplement: S4 Fig — A, B & C) ISG induction in the vagina, uterus and ovary in DMPA treated, uninfected, IFNε-/- mice following 6 h iVag treatment with the indicated IFN or buffer control. (TIF) [file ppat.1010843.s004.TIF]

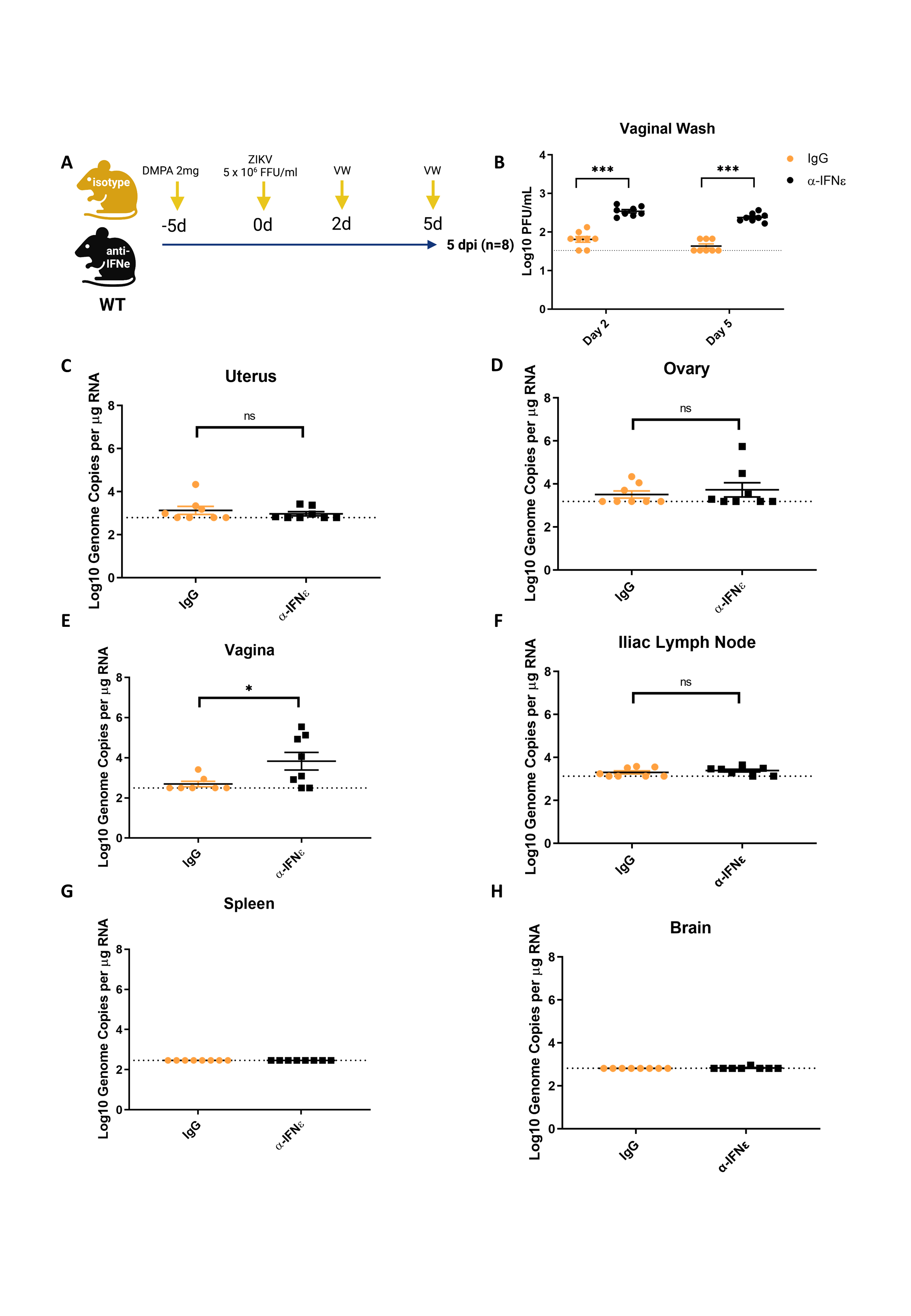

Supplement: S5 Fig — A) Experimental time line of (WT mice were treated 6 h prior to infection with 100 ug anti-IFNε or isotype control, then infected with ZIKV at 5 X10^5 FFU 5 days post DMPA treatment and vaginal washes were taken at 2, 5, mice were culled at 5 dpi. B) Infectious virus was measured from vaginal washes by plaque assay at 2 and 5 dpi. C, D, E, F, G) Tissues taken at 5 dpi were used to harvest RNA for analysis of viral RNA by qRT-PCR in the uterus, ovary, vagina, illiac lymph node spleen and brain respectively. Schematic created with BioRender.com. (TIF) [file ppat.1010843.s005.TIF]

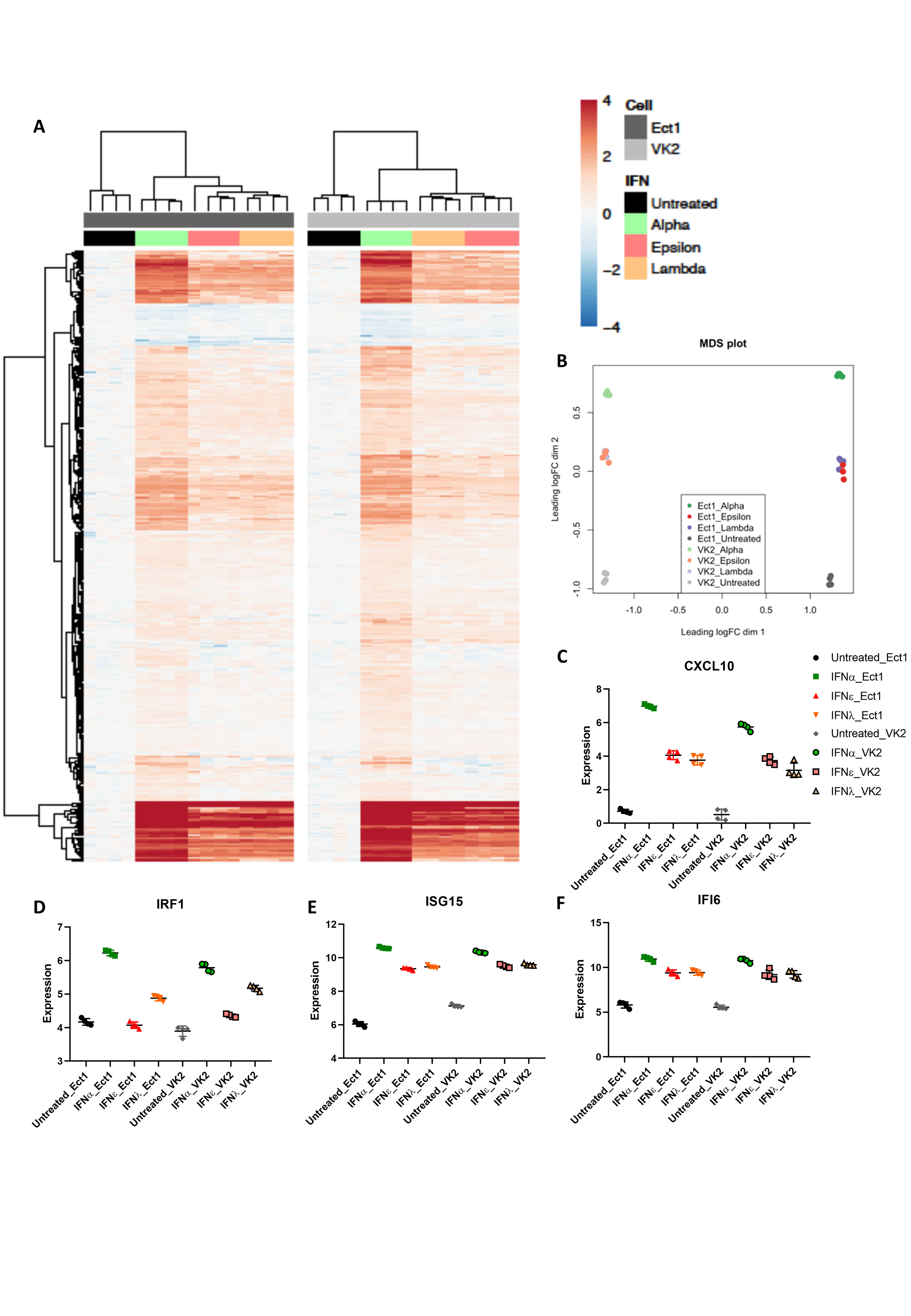

Supplement: S6 Fig — Differentially expressed genes were determined with a 1.2-fold cut-off and adjusted p-value < 0.05. A) Heat map showing expression of all differentially regulated genes. B) MDS plot showing the relationship between samples based on the top 500 most variable genes. C, D, E & F) Expression plots for CXCL10, IRF1, ISG15 and IFI6 respectively in Ect1 or VK2 cells. (TIF) [file ppat.1010843.s006.TIF]

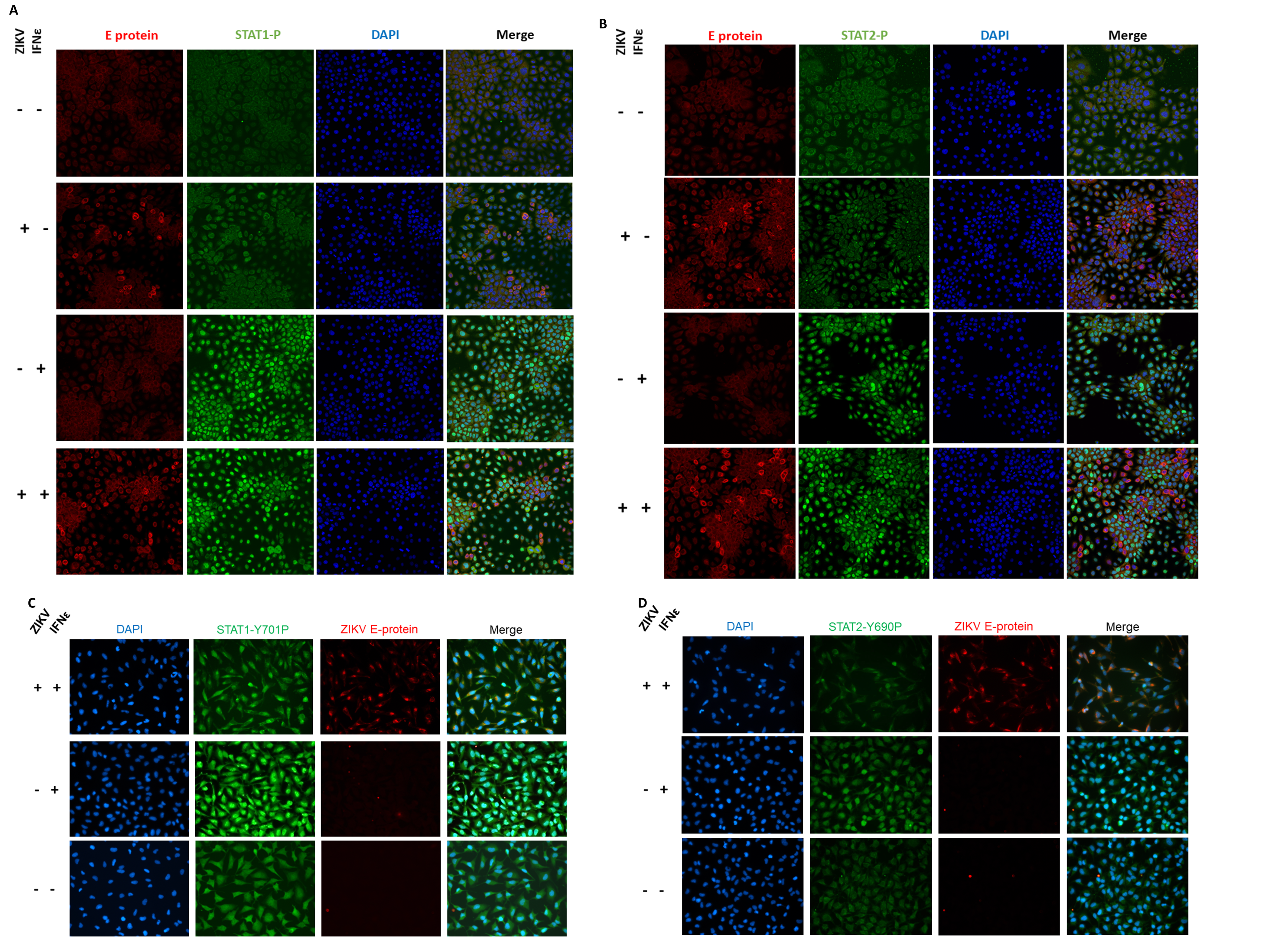

Supplement: S7 Fig — A & B) Ect1 cells were infected with ZIKV MOI of 10, 24h post infection cells were stimulated with hIFNε (100 U/mL) for 30 min then fixed with acetone/methanol for detection of ZIKV E antigen (Red) and phosphorylated STAT1 or STAT2 proteins (Green) by indirect immunofluorescence, DAPI (Blue). C & D) HeLa cells were infected with ZIKV MOI of 10, 24h post infection cells were stimulated with mIFNε (10 U/mL) for 30 min then fixed with acetone/methanol for detection of ZIKV E antigen (Red) and phosphorylated STAT1 or STAT2 proteins (Green) by indirect immunofluorescence, DAPI (Blue). (TIF) [file ppat.1010843.s007.tif]

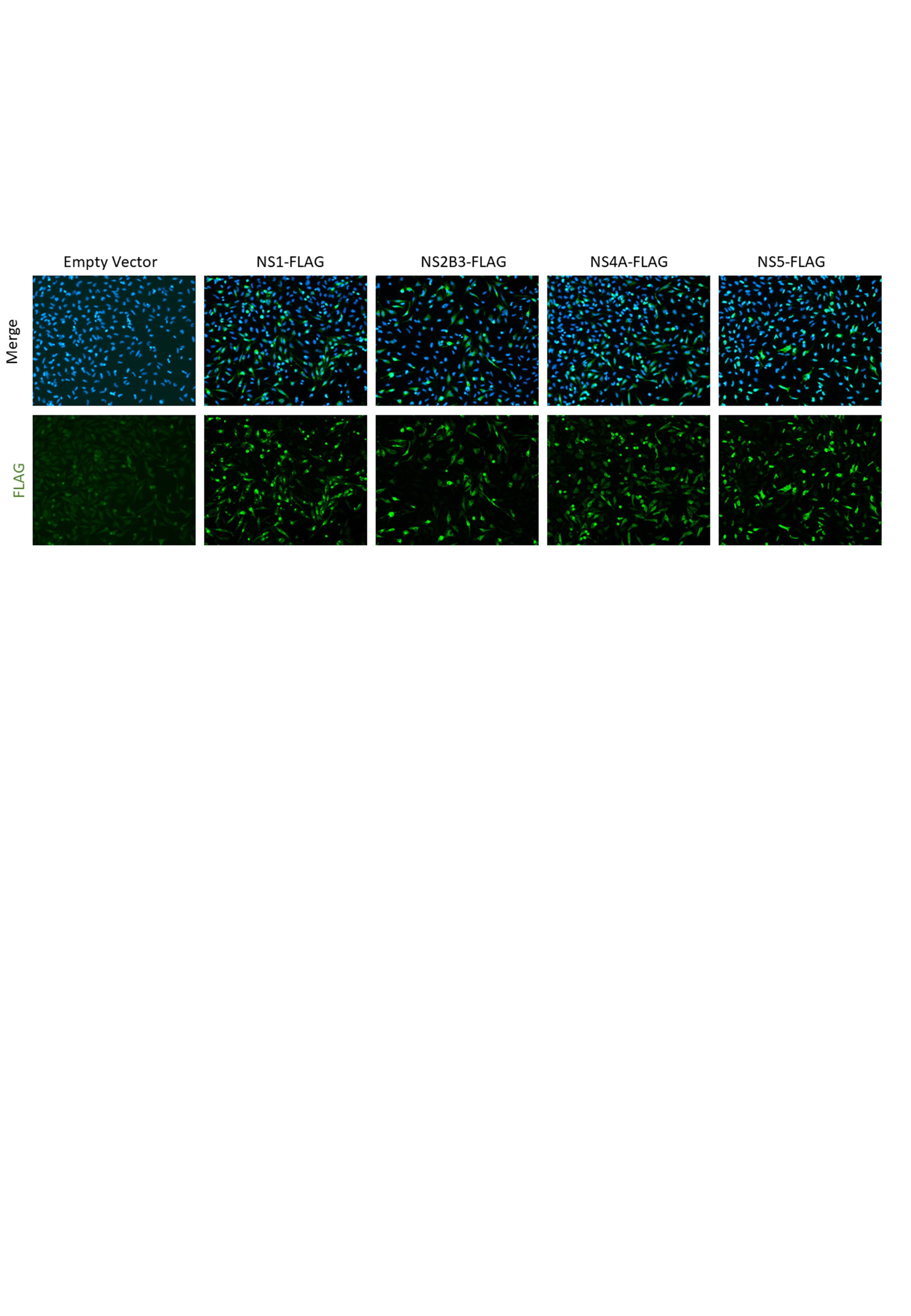

Supplement: S8 Fig — (TIF) [file ppat.1010843.s008.tif]

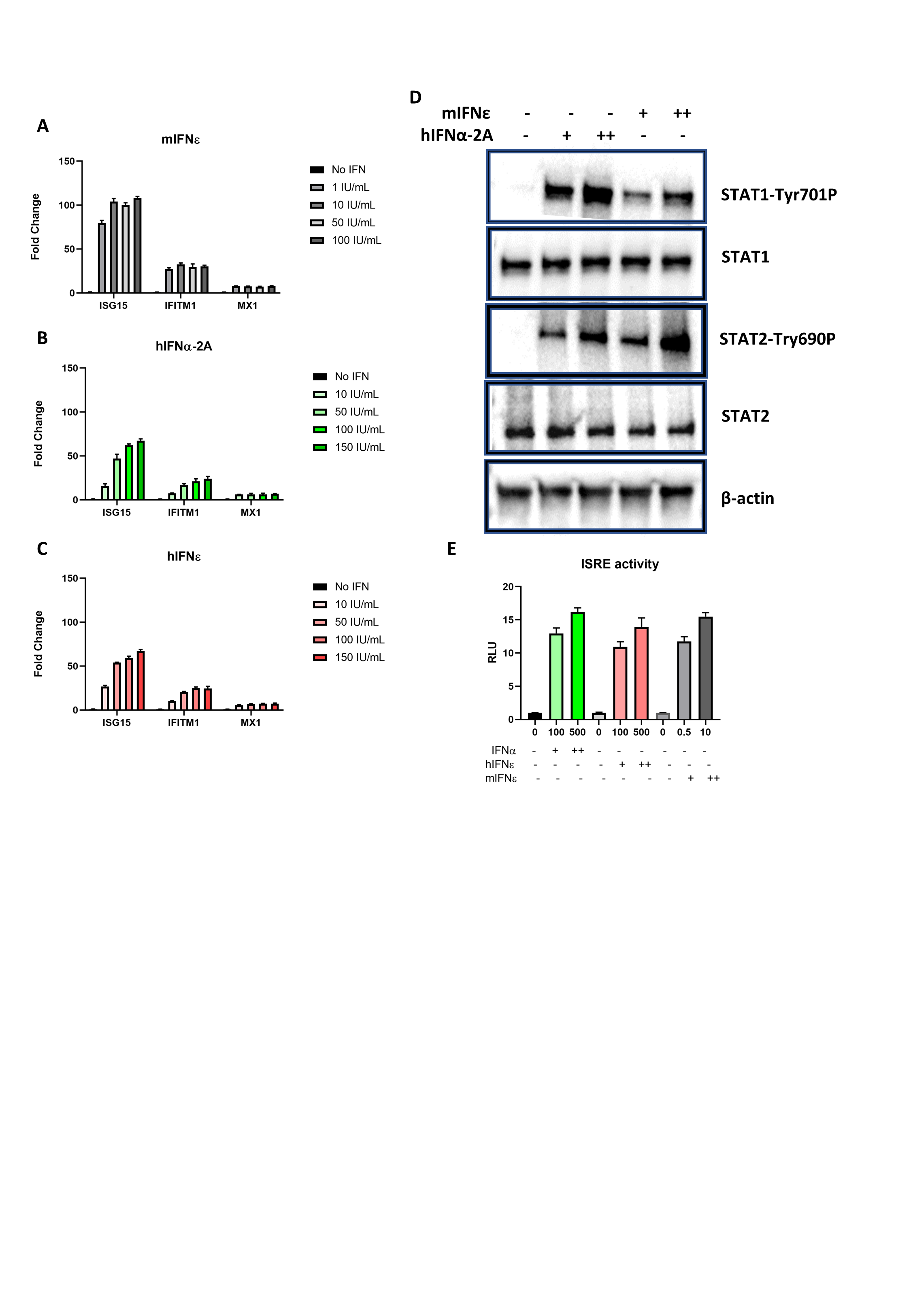

Supplement: S9 Fig — A, B & C) Dose dependent ISG induction in HTR8 cells following 6h stimulation with indicated amounts of type-I IFNs, data is expressed as fold change relative to untreated cells. D) Immunoblot of STAT1/STAT2 phosphorylation in HeLa cells 30 minutes post stimulation with mIFNε (0.5 or 10 U/mL) or hIFNαt-2A (100 or 500 U/mL). E) ISRE promoter activity in HeLa cells transfected with an ISRE luciferase reporter was measured by dual luciferase assay in relative light units (RLU) 8 h post stimulation with the indicated amounts of mIFNε. (TIF) [file ppat.1010843.s009.TIF]
