# Supplementary material for: Constitutive expression and distinct properties of IFN-epsilon protect the female reproductive tract from Zika virus infection
Source: PLoS Pathog. 2023 Mar 10;19(3):e1010843. doi: 10.1371/journal.ppat.1010843 (PMC10032502; doi:10.1371/journal.ppat.1010843)
Supplement: S1 Table — (DOCX) [file ppat.1010843.s010.docx]

**S1 Table. Antibodies used in this study.**

| **Primary Antibody** | **Dilution (IF)** | **Dilution (WB)** |
| --- | --- | --- |
| Mouse α - flavivirus E (4G2) hybridoma supernatant | 1/5 | NA |
| Chicken α – NS5 (in house) | NA | 1:1000 |
| Mouse α – FLAG (SIGMA M2) | 1:200 | 1:1000 |
| Rabbit α – STAT2-Y690P (CST#D3P2P) | 1:100 | 1:1000 |
| Rabbit α – STAT1-Y701P (CST#58D6) | 1:200 | 1:1000 |
| Rabbit α – STAT2 (CST#D9JL) | NA | 1:1000 |
| Rabbit α – STAT1 (CST#D1K9Y) | NA | 1:1000 |
