# Supplementary material for: Constitutive expression and distinct properties of IFN-epsilon protect the female reproductive tract from Zika virus infection
Source: PLoS Pathog. 2023 Mar 10;19(3):e1010843. doi: 10.1371/journal.ppat.1010843 (PMC10032502; doi:10.1371/journal.ppat.1010843)
Supplement: S2 Table — (DOCX) [file ppat.1010843.s011.docx]

**S2 Table. Primers used in this study**

| **Gene/Target** | **Sequence (5’ to 3’)** |
| --- | --- |
| 36B4 (mouse and human) | F – AGA TGC AGC AGA TCC GCA T  R – GGA TGG CCT TGC GCA |
| Human IFNe | F – TCA GCC TCT TCA GGG CAA ATA  R – GAG GAA TTT CTC CGT GTG GTT T |
| Human IFN-B | F – GCA GTC TGC ACC TGA AAA GAT ATT  R – TGT ACT CCT TGG CCT TGA GGT A |
| Human IFNL1 | F – GGA AGA GTC ACT CAA GCT GAA AAA C  R – AGA AGC CTC AGG TCC CAA TTC |
| Human IFNL2-3 | F – CAG CTG CAG GTG AGG GA  R – GCG GTG GCC TCC AGA ACC TT |
| human ISG15 | F – TGG CGG GCA ACG AAT T  R – GGG TGA TCT GCG CCT TCA |
| human IFIT1 | F – AAC TTA ATG CAG GAA GAA CAT GAC AA  R – CTG CCA GTC TGC CCA TGT G |
| human Viperin | F – GTG AGC AAT GGA AGC CTG ATC  R – GCT GTC ACA GGA GAT AGC GA |
| human CXCL10 (IP-10) | F – TCC ACG TGT TGA GAT CAT TGC  R – TCT TGA TGG CCT TCG ATT CTG |
| human CXCL11 | F – CCT TGG CTG TGA TAT TGT GTG C  R – CCA CTT TCA CTG CTT TTA CCC C |
| human IRF1 | F – CCA GCC CTG ATA CCT TCT CTG A  R – AAG TCC TGC ATG TAG CCT GGA A |
| human IFI6 | F – CTG AAG ATT GCT TCT CTT CTC  R – CAC TTT TTC TTA CCT GCC TC |
| Murine IFNE | F – GAA ACG GAT TCC CTT CCA AT  R – ACT GCT GGA CTG ACG AGC TT |
| Murine IFNA | F – CTG CCT GAA GGA CAG GAA GG  R – GTC ATT GAG CTG CTG GTG GA |
| Murine IFNB | F – AGA AAG GAC GAA CAT TCG GAA A  R – CCG TCA TCT CCA TAG GGA TCT T |
| Murine IFNL2 | F – CCA CAT TGC TCA GTT CAA GTC TCT  R – TCC TTC TCA AGC AGC CTC TTC T |
| murine ISG15 | F – GGG GCC ACA GCA ACA TCT AT  R – AGC CAG AAC TGG TCT TCG TG |
| murine IFIT1 | F – TGG CGT AGA CAA AGC TCT TCA TC  R – TAG CAG AGC CCT TTT TGA TAA TGT AA |
| murine Viperin | F – TTG GGC AAG CTT GTG AGA TTC  R – TGA ACC ATC TCT CCT GGA TAA GG |
| murine HPRT | F – AAG CTT GCT GGT GAA AAG GA  R – TTG CGC TCA TCT TAG GCT TT |
| murine CXCL10 | F – ATG ACG GGC CAG TGA GAA TG  R – ATG ATC TCA ACA CGT GGG CA |
| ZIKV PRVABC59 – prM specific | F – GTG TGA TGC CAC CAT GAG CTA  R – TGG CAG GTT CCG TAC ACA AAC |
